# Supervised resistance training in an individual with glioblastoma undergoing chemoradiation: a case report

**DOI:** 10.3389/fonc.2026.1711875

**Published:** 2026-01-21

**Authors:** Bruce Nakfoor, Ciaran M. Fairman

**Affiliations:** 1 University of Michigan Medical School, Ann Arbor, MI, United States; 2 Department of Exercise Science, Arnold School of Public Health, University of South Carolina, Columbia, SC, United States

**Keywords:** exercise oncology, glioblastoma, physical function, quality of life, resistance exercise

## Abstract

**Introduction:**

Exercise has demonstrated safety and efficacy in mitigating treatment-related symptoms across cancer populations; however, evidence in neuro-oncology remains limited, particularly during active chemoradiation.

**Methods:**

We report the case of a 63-year-old individual with newly diagnosed glioblastoma, experiencing symptoms of fatigue, balance issues, headaches, and memory loss, who initiated a 12-week supervised resistance training program during concurrent chemoradiation. Assessments at baseline and post-intervention included physical function and quality-of-life outcomes.

**Results:**

Adherence was 56% (20/36 sessions), and no adverse events occurred. The patient showed improvements in 6-minute walk distance and short physical performance battery score that exceeded established minimal clinically important differences. Quality-of-life findings were mixed, with certain domains benefiting from the exercise intervention and others worsening.

**Discussion:**

This case highlights the feasibility and safety of supervised resistance training during active glioblastoma chemoradiation. The observed improvements in physical function and select quality-of-life domains represent exploratory signals that compare favorably with the extant exercise oncology literature.

## Highlights

- Supervised resistance training appeared feasible and safe for this individual with glioblastoma during chemoradiation.
- The patient showed exploratory improvements in physical function and select psychosocial domains, alongside deterioration in several quality-of-life domains.
- Findings should be viewed as preliminary signals consistent with prior exercise-oncology literature.
- Larger, controlled studies are needed to determine whether exercise meaningfully affects outcomes during active treatment.

## 1 Introduction

Glioblastoma (GBM) is an aggressive primary brain tumor with a poor prognosis despite standard-of-care therapy. The current standard Stupp protocol for newly diagnosed GBM involves maximal surgical resection followed by concurrent radiotherapy with temozolomide and adjuvant temozolomide chemotherapy (1). Even with this intensive multimodal treatment, median survival remains under 2 years (2). GBM patients often experience severe treatment-related side effects, including fatigue, cognitive impairment, neurologic deficits, and muscle wasting, which can diminish functional status and quality of life (1, 3, 4).

Exercise is a well-recognized supportive care intervention in oncologic care, demonstrated to improve fatigue, fitness, and quality of life in many cancer types (5–12). In glioblastoma patients, preliminary studies have indicated that structured exercise is feasible and may reduce fatigue while enhancing quality of life (13–21). However, the majority of studies are performed in the rehabilitative setting after treatment is complete (22, 23), with exercise seldom offered to GBM patients during active treatment (15, 24). As a result, there is a gap in guidelines and practice regarding exercise for neuro-oncology patients, especially during concurrent chemoradiation treatment. Given the paucity of randomized evidence regarding exercise during active treatment, carefully documented case studies are valuable for illustrating feasibility and safety in this vulnerable population, as there may be an opportunity for early intervention during their treatment. Such reports can generate hypotheses and highlight directions for future research in neuro-oncology.

This case report describes a 63-year-old man with GBM who completed a supervised resistance training program during concurrent chemoradiation. The patient’s baseline characteristics, exercise prescription, and pre- and post-intervention outcomes on physical function and quality of life are detailed.

## 2 Case description

### 2.1 Individual history/diagnosis

The individual, a 63-year-old man without significant comorbid conditions, presented in September 2024 with new-onset generalized seizures. Magnetic resonance imaging (MRI) demonstrated a mass lesion in the right temporal lobe concerning a glial neoplasm. Following craniotomy (tumor resection and biopsy), the patient was diagnosed with a WHO Grade 4 glioblastoma with histopathologic features of IDH-1/2 wild type, TERT positive, EGFR positive, and MGMT promoter unmethylated. Staging evaluation was negative for distant metastases. An MRI performed following surgery showed residual disease, and the patient was initiated on standard concurrent chemoradiation (1). The patient had no family history of cancer and was negative for inherited genetic mutations. Prior to diagnosis, the patient self-reported engaging in a moderate level of physical activity.

### 2.2 Physical examination and baseline function

The post-surgical course was uncomplicated. At approximately 4 weeks following surgery (around initial chemoradiation), the patient was medically cleared to engage in moderate-intensity exercise with physician approval. The patient’s main self-reported symptoms prior to the exercise program were fatigue, episodic headaches, insomnia, balance disturbances, and memory changes, which are consistent with the normal clinical presentation of GBM patients starting their treatment course (1, 3, 4).

### 2.3 Treatment regimen

The patient began chemoradiation with local brain radiotherapy (60 Gy total over 6 weeks) with concurrent once-daily temozolomide (150 mg). He additionally took 1 mg of dexamethasone daily during treatment. He was started on levetiracetam 500 mg twice a day for seizure prophylaxis. Exercise training started in the second week of concurrent chemoradiotherapy and continued through the duration of treatment and into the adjuvant temozolomide treatment.

### 2.4 Exercise protocol

The patient was a part of a larger trial assessing the effects of resistance exercise in individuals treated for cancer (Advarra IRB: Pro00079265), and the resistance exercise intervention was delivered by trained study staff with prior experience in exercise oncology. Written informed consent was obtained prior to beginning the program. Exercise sessions were conducted at a dedicated exercise oncology facility affiliated with a radiation oncology practice in Southwest Florida.

The patient completed a 12-week supervised resistance training program, with three scheduled sessions per week on non-consecutive days. Each session lasted approximately 45 minutes and was delivered in a small group format with an instructor-to-participant ratio of 1:3–5. The program targeted fundamental movement patterns, including upper body pushing and pulling in vertical and horizontal planes, lower body hinging and squatting, and core strengthening movements. This approach has been used successfully in our and others’ prior work in exercise oncology (25–34).

Exercise selection and program modifications were individualized based on the participant’s movement competency, pre-existing impairments, and clinical presentation. Study staff assessed his performance and symptoms throughout each session and adjusted exercises as necessary to ensure safety and appropriateness. The load prescription used a self-determined repetitions in reserve (SD RIR) approach (25, 26). During an initial familiarization week, the patient was taught the SD RIR concept and completed anchoring exercises to calibrate perceived exertion. For each exercise, he selected a load that permitted the prescribed repetitions while leaving the target number of repetitions in reserve. When he consistently completed the prescribed number of repetitions at a given load, the load was incrementally increased while maintaining the target repetition range (25–28). Conversely, if he showed excessive fatigue or symptom exacerbation, the load was reduced, or the exercise was regressed. Prior to each exercise session, the patient scored his energy, pain, and sleep quality from the previous night (**Table 1**). The patient’s full exercise program can be found in **Supplementary Material**.

**Table 1:** Patient’s sleep, energy, and pain scored prior to each exercise session.

Workout #	Sleep (0–10)	Energy (0–10)	Pain (0–10)
1	6	7	0
2	5	9	1
3	5	10	0
4	X	X	X
5	5	8	0
6	6	8	0
7	X	X	X
8	5	10	0
9	5	9	0
10	6	9	0
11	7	10	0
12	7	10	0
13	X	X	X
14	X	X	X
15	X	X	X
16	X	X	X
17	X	X	X
18	X	X	X
19	7	10	0
20	7	10	0
21	X	X	X
22	7	10	0
23	8	10	0
24	X	X	X
25	8	10	0
26	8	10	0
27	X	X	X
28	8	10	0
29	8	10	0
30	8	10	0
31	9	10	0
32	9	10	0
33	X	X	X
34	X	X	X
35	X	X	X
36	X	X	X

X denotes missed session.

**Figure 1:**
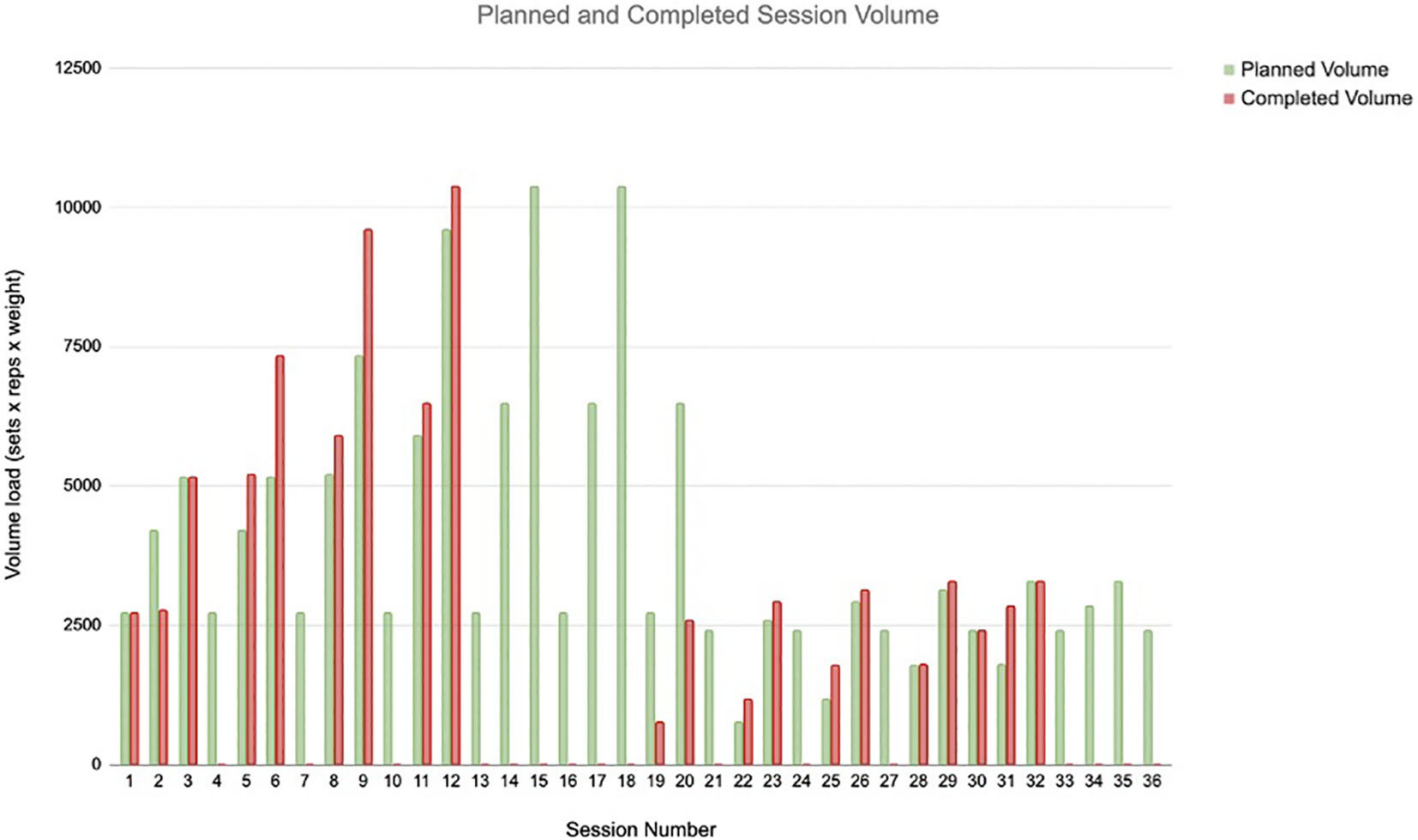
Patient exercise relative dose intensity. *The reduction in training volume observed from session 19 onward was attributable to modifications in the exercise program implemented after week 6. Beginning at session 19, higher-load, multi-joint lifts such as the hex bar squat and hip thrust were swapped in the program for variety, resulting in lower overall training volume during sessions 19–36.

Each exercise session had a prescribed target resistance training volume (sets × repetitions × load for each exercise). Exercise dose was operationalized using the relative dose intensity (RDI) method, which quantifies the proportion of the prescribed exercise dose that is actually completed (28). For each session, total training volume was calculated as the sum of sets × repetitions × load across all exercises, and RDI was computed as (completed volume ÷ prescribed volume) × 100, expressed as a percentage. Session-specific RDI values for this participant across the intervention are presented in **Figure 1**; missed sessions were assigned an RDI of 0%. Outside of supervised sessions, he was encouraged to remain physically active within his comfort level through activities such as walking or cycling.

## 3 Outcome assessments

Before the start of the exercise intervention, the patient underwent a comprehensive baseline assessment battery.

### 3.1 Physical function

The short physical performance battery (SPPB) and 6-minute walk test (6MWT) were the primary physical function assessments. The SPPB is an objective assessment of lower extremity function, and the test involves three components: 1) balance, evaluated with side-by-side, semi-tandem, and tandem stances (held for up to 10 seconds each); 2) chair sit-to-stand (the time required to complete five repetitions with arms crossed); and 3) gait speed (the average time to walk 4 m at a usual pace, with the faster of two trials recorded) (35, 36). Each component is scored 0–4, with higher values indicating greater functional capacity, and then summed (total 0–12).

The 6MWT measures the total distance that a patient can walk for 6 minutes on flat ground and is an indicator of submaximal aerobic capacity (37, 38). For this test, a walking course was created at the exercise facility in which the patient could walk in a straight line on a flat surface for 15 m, prior to turning around a cone, and returning to the starting position. The participant was instructed to walk as quickly as possible, without running. A chair was available close to the walkway if the participant experienced fatigue.

### 3.2 Cancer-related fatigue

Cancer-related fatigue was measured using the 13-item Functional Assessment of Chronic Illness Therapy - Fatigue Scale (FACIT-Fatigue). Each item asks how true a statement about fatigue was “over the past 7 days”, rated on a 5-point Likert scale from 0 (not at all) to 4 (very much) (39–41). The scores are summed for a total range of 0–52, with higher scores denoting less fatigue.

### 3.3 Quality of life

Quality of life was assessed using the 36-item Short Form Health Survey (SF-36) (42–44). The SF-36 covers eight domains: physical functioning, role limitations due to physical health, bodily pain, general health perceptions, energy/fatigue (vitality), social functioning, role limitations due to emotional problems, and emotional well-being. Each domain is scored by recoding its item responses to a 0–100 scale (0 = worst health, 100 = best) and averaging the items in that domain. A higher SF-36 score indicates a better quality of life.

## 4 Observations and outcomes

**Figure 2:**
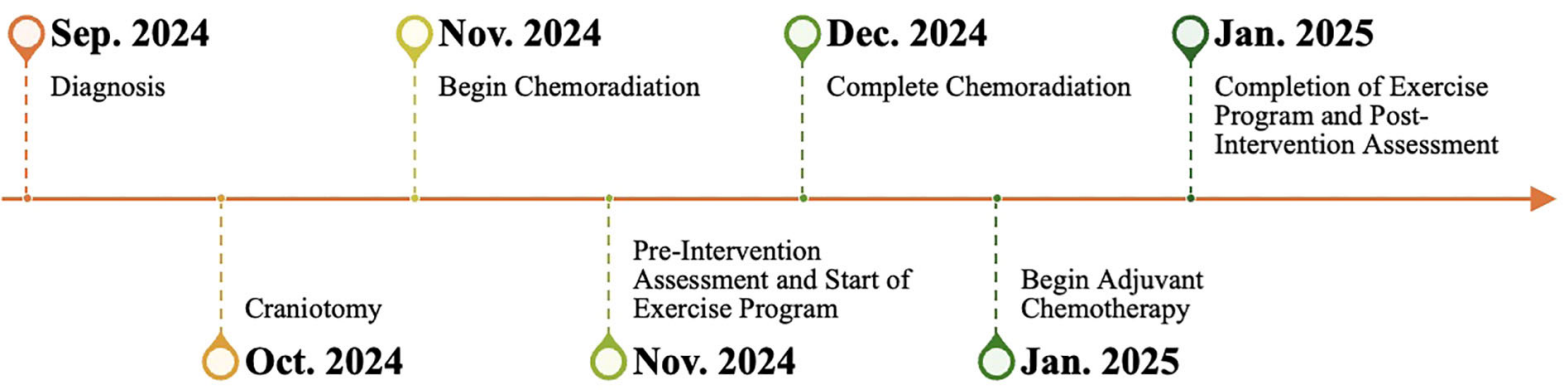
Timeline of patient care.

The patient completed the initial 6-week course of radiation and concurrent daily temozolomide treatment by the fifth week of the supervised exercise intervention. He continued exercise sessions while transitioning to adjuvant temozolomide cycles. The exercise intervention was completed at 12 weeks, shortly after he completed his first adjuvant chemotherapy cycle. **Figure 2** presents a timeline of the patient’s care, detailing the sequence of his primary surgery, subsequent treatment phases, the initiation and progression of supervised resistance training sessions, and the timing of all clinical and functional assessment time points. The patient’s baseline and post-intervention testing results are shown in **Table 2**.

**Table 2:** Pre- and post-intervention physical function and quality-of-life scores.

Physical function	Pre	Post	Change
SPPB			
Balance	4	4	0
Gait speed	4	4	0
Sit to stand	2	4	2
Total	10	12	2
6-Minute walk test	600	630	30
FACIT-F	43	50	+7
SF-36			
Physical functioning	95	100	+5
Role limitations due to physical health	50	0	−50
Role limitations due to emotional problems	66.7	100	+33.3
Energy/fatigue	90	85	−5
Emotional well-being	76	96	+20
Social functioning	100	87.5	−12.5
Pain	77.5	77.5	0
General health	80	75	−5
Health change	50	50	0

SPPB, short physical performance battery; SF-36, 36-item Short Form Health Survey.

### 4.1 Physical performance

The patient’s SPPB score improved from 10 to 12, driven by gains in sit-to-stand performance (+2 points), while balance and gait speed remained stable. His 6MWT increased by 30 m (from 600 to 630 m).

### 4.2 Exercise adherence and intensity

RDI was tracked across sessions (**Figure 1**). Exercise volume decreased in the second phase of training (sessions 19–36) after protocol modification to reduce higher-load lifts (e.g., hex bar squat and hip thrust) and replace them with lower load exercises (dumbbell Romanian deadlift, dumbbell step up, and single-leg stance variations). Higher load lifts, such as the hex bar squat and hip thrust, permit substantially greater external loads for a given relative intensity. In contrast, exercises performed with a single-leg stance or reduced base of support require a challenging effort at lighter absolute loads. An adequate training stimulus was therefore maintained, but the lighter absolute loads for certain exercises resulted in a lower session volume across the latter half of the 12-week program. Of 36 scheduled sessions, 16 were missed, primarily due to conflicting appointments (n = 7) or illness (n = 3).

### 4.3 Patient-reported outcomes

The participant reported improved psychological well-being at program completion compared with baseline. FACIT-Fatigue score increased by 7 points (Pre = 43; Post = 50). Based on the SF-36 scores, the participant’s emotional well-being improved (Pre = 76; Post = 96), as well as role limitations due to emotional problems (Pre = 66.7; Post = 100). In contrast, general health (Pre = 80; Post = 75) and energy/fatigue (Pre = 90; Post = 85) declined slightly. Physical functioning improved (Pre = 95; Post = 100), but role limitations due to physical health worsened (Pre = 50; Post = 0). Pain (77.5) and health change (50) remained the same, while social functioning declined (Pre = 100; Post = 85). Pre-exercise self-reports showed improved sleep (from 5–6/10 to 8–9/10 by later sessions), consistently high energy (stabilizing at 10/10 from session 11 onward), and negligible pain (only one report of 1/10 across all sessions) (**Table 1**).

## 5 Discussion

This case study illustrates the feasibility and safety of integrating a supervised resistance training program into the care of a patient with glioblastoma undergoing active treatment. The patient tolerated moderate-to-vigorous resistance training alongside concurrent chemoradiation and demonstrated improvements in several health domains. Importantly, the patient completed the 12-week training intervention without adverse events, consistent with findings from larger neuro-oncology exercise studies (15). Although patients with brain cancer are at risk for seizures, falls, and sudden neurologic changes during exertion, this case suggests that such risks can be effectively managed with appropriate pre-participation screening and close supervision.

Adherence to the exercise program was moderate, with the patient completing ~56% of scheduled sessions during concurrent chemoradiation. Missed sessions were due to conflicting medical appointments, supplementary clinical consultations, a viral illness, and a brief vacation. Prior exercise-oncology studies have similarly shown that tailored programs improve compliance, although adherence rates in individuals with brain tumors remain highly variable (33%–100%) even under supervised conditions (16, 18, 19, 45, 46). Within this context, the patient’s sustained participation during chemoradiation and transition into adjuvant therapy supports the feasibility of structured exercise in individuals with glioblastoma when adequate support is provided.

The patient also demonstrated clinically meaningful improvements in the 6MWT and the SPPB, which is promising, particularly given that the 6MWT and SPPB have previously been reported to decline significantly during chemotherapy in individuals with cancer (47–49). The 6MWT distance increased by 30 m (Pre = 600 m; Post = 630 m), which falls within the established minimal clinically important difference (MCID) of 20–50 m reported in oncology and older adult populations, where reductions of this magnitude are associated with an increased risk of mortality (50–53). Similarly, the SPPB scaled score improved from 10 to 12, exceeding the minimal clinically important difference of 1 point, where each 1-unit increase has been associated with an approximate 12% reduction in mortality in individuals with cancer, and scores below 10 are predictive of higher all-cause mortality (36, 54–56). Taken together, these MCID level improvements indicate a clinically relevant shift in physical function during chemoradiation, although the uncontrolled design of this case study precludes any conclusions around intervention efficacy.

The patient also reported changes in fatigue and health-related quality of life. FACIT-Fatigue increased by 7 points (Pre = 43; Post = 50), exceeding the minimal clinically important difference of 3 to 4 points in cancer populations (36), which is consistent with a meaningful reduction in cancer-related fatigue. On the SF-36, emotional well-being improved by 20 points (Pre = 76; Post = 96), and role limitations due to emotional problems by 33.3 (Pre = 66.7; Post = 100), both surpassing the typical 5–10 threshold for clinical relevance (57). These improvements occurred alongside clinically relevant deterioration in other domains. General health and vitality declined modestly (−5 points each), and role limitations due to physical health worsened from 50 to 0, despite stable or improved performance-based measures. In addition, social functioning decreased from 100 to 85, a pattern frequently observed during intensive oncologic care, where frequent visits, infection concerns, and chronic fatigue can restrict social participation (12, 58–60). The mixed pattern of improvement and deterioration in fatigue and quality-of-life (QOL) outcomes likely reflects the combined influence of treatment burden, evolving understanding of prognosis, and psychosocial stressors, in addition to any potential effect of the exercise program (58, 59, 61–63). In broader oncology populations, exercise interventions consistently alleviate fatigue and enhance quality of life, and clinical guidelines now strongly encourage patients to remain physically active during treatment (5, 64, 65). Taken collectively, these results suggest that structured exercise may have contributed to improvements in select psychosocial domains for this patient, although the single-case, uncontrolled design means that these findings should be interpreted as exploratory signals that align with broader exercise oncology literature rather than evidence of causality.

## 6 Limitations

Despite these positive findings, several limitations warrant consideration. As a single case report, the observations cannot be generalized beyond this individual with glioblastoma. The favorable outcomes likely reflect patient-specific factors such as relatively high baseline functional status, strong motivation, and preserved cognition; many people with glioblastoma have neurologic or cognitive deficits that require adapted exercise and may limit achievable improvements (66, 67). Thus, improvements in physical function and quality of life may be less pronounced in more impaired populations.

The uncontrolled design also introduces the potential for confounding. Changes in function and symptoms may have been influenced by concurrent oncologic treatment, natural recovery, unsupervised physical activity, medication changes, or greater clinical attention, so attribution of effects to the exercise program is not possible. Patient-reported outcomes (SF-36 and FACIT), while validated in cancer populations, are vulnerable to recall and response bias and were collected at a single post-intervention time point, limiting insight into trajectories and durability of change. Similarly, as the patient could not be reached at the time of manuscript preparation, we were unable to obtain a patient perspective, and the report reflects only the objective findings from the post-program assessment. Generalizability is further restricted by the context of supervised training with an exercise professional in a dedicated facility, which many individuals with brain tumors may not be able to access because of travel, financial, or time constraints. Overall, this case suggests that supervised exercise during chemoradiation is feasible and appears safe in selected patients, but potential benefits and appropriate candidates require confirmation in larger controlled studies.

Future research is needed to validate and expand upon these observations. Ongoing and planned randomized trials will be essential to determine the effectiveness of exercise in glioblastoma, define optimal training prescriptions (e.g., modality, intensity, and frequency), and identify patient subgroups most likely to benefit (3, 16, 68–70). Such evidence will be critical to inform clinical guidelines and support the integration of structured exercise into standard neuro-oncology care.

## 7 Conclusion

This case suggests that a 12-week supervised resistance training program was feasible and safe during chemoradiation in a patient with glioblastoma and was associated with improvements in physical performance and select quality-of-life domains. Although these findings are limited to a single individual, they are consistent with emerging evidence supporting the role of structured exercise as a feasible and potentially beneficial supportive care strategy in neuro-oncology.

## 8 Patient perspective

A patient perspective could not be included, as the patient could not be contacted at the time of manuscript preparation. Although anecdotally, the patient reported having a positive experience with the program, we were unable to characterize his longer-term experience after program completion. This limits the depth of qualitative context in this case study and should be considered a limitation. Future research in exercise interventions in neuro-oncology should incorporate a structured collection of patient perspectives at multiple time points, including after the intervention has concluded.

## Data Availability

The original contributions presented in the study are included in the article/**Supplementary Material**. Further inquiries can be directed to the corresponding author.
